# Occlusal conditions, postural control and plantar parameters in adults and growing subjects: a systematic review of objective assessment methods

**DOI:** 10.3389/fdmed.2026.1887418

**Published:** 2026-07-09

**Authors:** Isabel Carda-Navarro, Ana Sanchez-Albero, Clara Guinot-Barona, Esther García-Miralles, Laura Marqués-Martínez, Juan Ignacio Aura-Tormos

**Affiliations:** 1 Faculty of Medicine and Health Sciences, Catholic University of Valencia, San Vicente Martir, Valencia, Spain; 2 Dentistry Department, Faculty of Medicine and Dentistry, University of Valencia, Valencia, Spain

**Keywords:** dental malocclusion, plantar pressure, postural control, posturography, systematic review

## Abstract

**Objective:**

To evaluate whether occlusal conditions are associated with global postural control and plantar parameters when assessed using objective posturographic and pressure-based methods, with particular attention to differences between adult populations and growing subjects.

**Methods:**

A systematic review was conducted following PRISMA 2020 guidelines. Searches were performed in PubMed/MEDLINE, Scopus, Embase, CINAHL, and Web of Science up to October 2025. Observational and experimental studies in humans assessing occlusal conditions in relation to instrument-based postural or plantar outcomes were included. Study selection, data extraction, and risk of bias (JBI) were conducted independently by two reviewers. Due to heterogeneity, findings were synthesized qualitatively. Certainty of evidence was evaluated using GRADE.

**Results:**

Seven studies were included. Outcomes comprised center-of-pressure metrics, postural sway, weight distribution, plantar pressure patterns, and balance performance. In adults, findings were inconsistent: under natural static conditions one study found no association while another reported significant static differences, and the remaining positive findings emerged mainly under experimental or task-specific conditions. The two studies in growing subjects yielded conflicting results (one larger study largely null, one positive), so the evidence does not support a more consistent association in this subgroup. Risk of bias related mainly to confounding and participant selection, and certainty of evidence was low to very low.

**Conclusions:**

Current evidence does not support a consistent relationship between occlusal conditions and global postural control. The two studies in growing subjects yielded conflicting findings, and the small number of studies, methodological heterogeneity, and low to very low certainty of the evidence preclude establishing a pediatric association or drawing firm clinical inferences.

**Systematic Review Registration:**

https://www.crd.york.ac.uk/prospero/display_record.php?RecordID=1266834, PROSPERO CRD420251266834.

## 1 Introduction

The human postural system is a highly integrated functional unit resulting from continuous interactions between musculoskeletal structures, neuromuscular control mechanisms, and multisensory input, including visual, vestibular, and proprioceptive information. Rather than representing a static condition, postural stability is a dynamic process that requires continuous adaptation to maintain balance during both standing and locomotion. Alterations in sensory processing or biomechanical constraints may therefore lead to measurable changes in postural sway and center-of-pressure behavior, reflecting the complexity of postural regulation mechanisms (1–3).

Within this framework, the role of the foot–ground interface has received particular attention, as plantar sensitivity and pressure distribution provide critical afferent input for postural control. Both static and dynamic plantar assessments have been widely used to evaluate balance and gait, and variations in plantar loading patterns have been associated with changes in postural stability across different populations (4–6). These findings support the concept that postural control emerges from the integration of multiple peripheral and central inputs rather than from isolated anatomical structures.

Traditionally, the stomatognathic system—comprising the teeth, jaws, temporomandibular joints, and associated musculature—has been considered functionally independent from global postural regulation. However, this compartmentalized view has been increasingly challenged. It has been proposed that occlusal conditions may influence postural control through neuromuscular interactions involving the masticatory and cervical musculature, as well as central postural control pathways (7). Variability related to age, sex, and cervical alignment further complicates the interpretation of postural outcomes and underscores the need for controlled and methodologically robust investigations (8).

Occlusal conditions, including dental malocclusion and experimentally induced occlusal alterations, are highly prevalent and clinically relevant across all age groups (9, 10). Beyond their well-established implications within the oral cavity, these conditions have been hypothesized to contribute to extraoral adaptations involving head posture, cervical alignment, and global postural control. This hypothesis is supported by the anatomical and functional continuity between the stomatognathic system and adjacent neuromuscular structures, which may facilitate the transmission of occlusal changes to the postural system.

In this context, several experimental and observational studies have explored the relationship between occlusal conditions and postural or plantar outcomes. Reported parameters include changes in center-of-pressure displacement, plantar pressure distribution, weight-bearing asymmetry, and balance performance under different occlusal conditions, such as clenching or experimentally induced occlusal alterations (11–13). However, findings remain inconsistent. While some studies have reported statistically significant associations, others have failed to identify meaningful relationships, even when similar outcome measures were employed (14, 15). Importantly, several authors have suggested that the influence of occlusal conditions on postural control may depend on age, neuromuscular maturation, and specific experimental testing conditions, rather than representing a uniform phenomenon across populations (16).

A major limitation of the existing literature lies in the marked heterogeneity of study designs, populations, definitions of occlusal conditions, and methods used to assess posture and plantar function. Differences between static and dynamic assessments, variability in outcome measures, and limited sample sizes hinder meaningful comparisons and reduce the strength of the available evidence. In addition, previous reviews have often combined heterogeneous methodologies without specifically focusing on objective posturographic and plantar pressure assessments, limiting the interpretability of their conclusions (17–19).

This question is particularly relevant during childhood and adolescence. Craniofacial growth, the transition through the mixed and early permanent dentition, and the maturation of balance, gait, and musculoskeletal function proceed in parallel, while the developing postural system continues to refine the integration of visual, vestibular, and proprioceptive input (1, 3). If occlusal conditions interact with postural control, such interactions might be more readily detectable while these systems are still developing, before adult compensatory mechanisms are fully established. Clarifying whether occlusal conditions are associated with measurable postural or plantar changes in growing subjects is therefore relevant to pediatric dentistry and interceptive orthodontics, where diagnostic and treatment decisions are made during active growth (19).

From an orthodontic and pediatric dentistry perspective, understanding whether these conditions—particularly sagittal discrepancies during growth—are associated with postural adaptations may have implications for diagnosis and interdisciplinary decision-making, but should not be interpreted as evidence that malocclusion is an independent cause of postural dysfunction or that occlusal treatment can improve posture.

Given the increasing interest in interdisciplinary approaches involving dentistry, physiotherapy, and podiatry, a focused synthesis of the available evidence using objective assessment methods is warranted. Therefore, the aim of this systematic review was to evaluate whether occlusal conditions are associated with postural or plantar parameters assessed using objective posturographic and plantar pressure methods, with particular attention to differences between adult populations and growing subjects.

## 2 Material and methods

### 2.1 Study design and reporting guidelines

This systematic review was conducted in accordance with the Preferred Reporting Items for Systematic Reviews and Meta-Analyses (PRISMA) 2020 guidelines (20). The review protocol was defined *a priori* and prospectively registered in PROSPERO (CRD420251266834). The protocol specified the eligibility criteria, information sources, search strategy, study selection process, data extraction procedures, and planned approach for risk of bias assessment and data synthesis, in order to minimize methodological bias and ensure transparency and reproducibility.

### 2.2 Eligibility criteria

Eligibility criteria were established *a priori* according to the Population–Exposure–Outcome (PEO) framework, which is appropriate for reviews evaluating associations rather than therapeutic interventions. The population comprised human participants of any age or sex, including both healthy individuals and participants with dental malocclusion or occlusal conditions, provided that no neurological, vestibular, or musculoskeletal disorders likely to interfere with postural assessment were reported. For subgroup interpretation, growing subjects were defined as children or adolescents in whom craniofacial growth and postural maturation were not yet complete, typically corresponding to the mixed or early permanent dentition. Exposure was defined as the presence of dental malocclusion or occlusal conditions, including sagittal, vertical, or transverse malocclusions, occlusal asymmetries, or experimentally induced occlusal alterations. Eligible outcomes included objective postural or plantar variables, such as body posture, postural sway, center-of-pressure displacement, plantar pressure distribution, balance performance, or gait-related parameters assessed under static and/or dynamic conditions.

Original research studies employing observational (cross-sectional, case–control, or cohort) or experimental designs were considered eligible. Studies were excluded if they were conducted in animals or *in vitro* models, focused exclusively on temporomandibular disorders without assessment of postural or plantar outcomes, lacked objective postural measurements, such as stabilometric, posturographic, plantar pressure, gait, or other validated quantitative assessment methods, or constituted secondary research (systematic reviews, narrative reviews), case reports, editorials, or conference abstracts.

### 2.3 Information sources and search strategy

A comprehensive electronic search was conducted in PubMed/MEDLINE, Scopus, Web of Science, Embase, and CINAHL, covering all records from database inception to October 2025. Additionally, no time-based filter was applied and only articles published in English were included. This restriction was applied for feasibility reasons related to study screening, full-text assessment, and data extraction. The search strategy followed a structured Boolean approach combining terms related to dental malocclusion and occlusal conditions with terms related to postural assessment. Specifically, keywords referring to malocclusion or dental occlusion were combined using the operator “OR” and subsequently linked via the operator “AND” to terms related to posture, postural control, plantar pressure, center of pressure, balance, and gait. Both controlled vocabulary terms and free-text keywords were used when applicable to maximize sensitivity. This general Boolean strategy was adapted for each database to account for differences in indexing systems and search syntax. In addition, the reference lists of all included studies and relevant review articles were manually screened, and citation tracking was performed to identify further eligible publications.

The full electronic search strategies for each database are provided in Supplementary Material 2.

### 2.4 Study selection process

All retrieved records were imported into reference management software, and duplicate entries were removed prior to screening. Two reviewers independently screened titles and abstracts to identify potentially eligible studies. Full texts of all records deemed relevant by at least one reviewer were subsequently assessed for eligibility according to the predefined criteria. Disagreements at any stage of the selection process were resolved through discussion and consensus; when necessary, a third reviewer was consulted. Inter-reviewer agreement during the full-text screening stage was assessed using Cohen's kappa coefficient, demonstrating excellent agreement (*κ* = 0.82).

Records were managed using Zotero reference management software (Corporation for Digital Scholarship, Vienna, VA, USA), and the screening process was performed using Rayyan (Qatar Computing Research Institute, Doha, Qatar).

### 2.5 Data extraction

Data extraction was performed independently by two reviewers using a predefined and piloted data extraction form. Extracted information included bibliographic details, study design, population characteristics, age group, chronological age, dentition stage when reported, developmental status when available, type and classification of malocclusion, distinction between natural malocclusion and experimentally induced occlusal alterations, testing condition, postural and/or plantar assessment methods, and main outcomes. To minimize extraction errors and ensure consistency, a random sample of 20% of the included studies was re-extracted independently by both reviewers, achieving a concordance rate greater than 95%. Any discrepancies were resolved through discussion until full consensus was reached.

### 2.6 Risk of bias assessment

Risk of bias was independently assessed by two reviewers using the Joanna Briggs Institute (JBI) Critical Appraisal Checklists, selecting the appropriate tool according to each study design (analytical cross-sectional or quasi-experimental studies, when applicable).

Each domain was rated as “yes”, “no”, “unclear”, or “not applicable”, in accordance with JBI guidance. Particular attention was given to domains related to participant selection, measurement of exposure and outcomes, identification and management of confounding factors, and appropriateness of statistical analysis. In addition to the domain-level JBI assessment, an overall qualitative judgement was assigned to each study. For the purpose of the overall qualitative judgement, studies were classified as low risk of bias when concerns were limited to isolated domains and no major methodological weaknesses were identified in key areas such as participant selection, confounding factors, exposure measurement, outcome assessment, or statistical analysis. Studies were classified as moderate risk of bias when concerns were identified in one or more key domains that could potentially influence the interpretation of the findings. Studies were classified as high risk of bias when multiple key domains presented important methodological limitations likely to compromise the validity of the results. These decision rules were defined *a priori* by the review team to improve transparency and reproducibility and were intended solely to support narrative interpretation rather than to generate a quantitative JBI score.

Disagreements between reviewers were resolved through discussion and consensus. The results of the risk-of-bias assessment were summarized in tabular form, with individual studies presented in rows and appraisal domains across columns.

The STROBE (Strengthening the Reporting of Observational Studies in Epidemiology) statement was used solely to describe reporting completeness and transparency, and was not considered a tool for assessing methodological quality or risk of bias.

### 2.7 Data synthesis

Due to substantial heterogeneity in study designs, participant characteristics, developmental stage, definitions of occlusal conditions, posturographic and plantar pressure assessment protocols, testing conditions (static vs. dynamic), and outcome measures, quantitative meta-analysis was not considered appropriate. A structured narrative synthesis was therefore conducted.

Where possible, findings were grouped according to population (adult vs. growing subjects), type of occlusal condition (natural malocclusion vs. experimentally induced alterations), and assessment context (static vs. dynamic conditions), allowing identification of consistent patterns and potential sources of heterogeneity across studies. When reported, interpretation also considered whether statistically significant findings were accompanied by information on effect magnitude or clinical relevance. In the absence of predefined thresholds or clinically interpretable effect estimates, statistically significant findings were not assumed to be clinically meaningful.

### 2.8 Certainty of evidence

The certainty of the evidence was assessed using the Grading of Recommendations, Assessment, Development, and Evaluations (GRADE) approach.

As all included studies were observational, the certainty of evidence started at a low level and was subsequently downgraded based on predefined domains, including risk of bias, inconsistency, indirectness, and imprecision. Publication bias was not formally assessed due to the limited number of included studies.

Because the available evidence comprised a small number of heterogeneous studies that differed substantially in terms of populations, occlusal exposures, assessment protocols, and outcome measures, the GRADE assessment was performed as an overall judgment of the body of evidence rather than separately for specific populations, exposure categories, or outcome domains.

## 3 Results

### 3.1 Study selection

**Figure 1:**
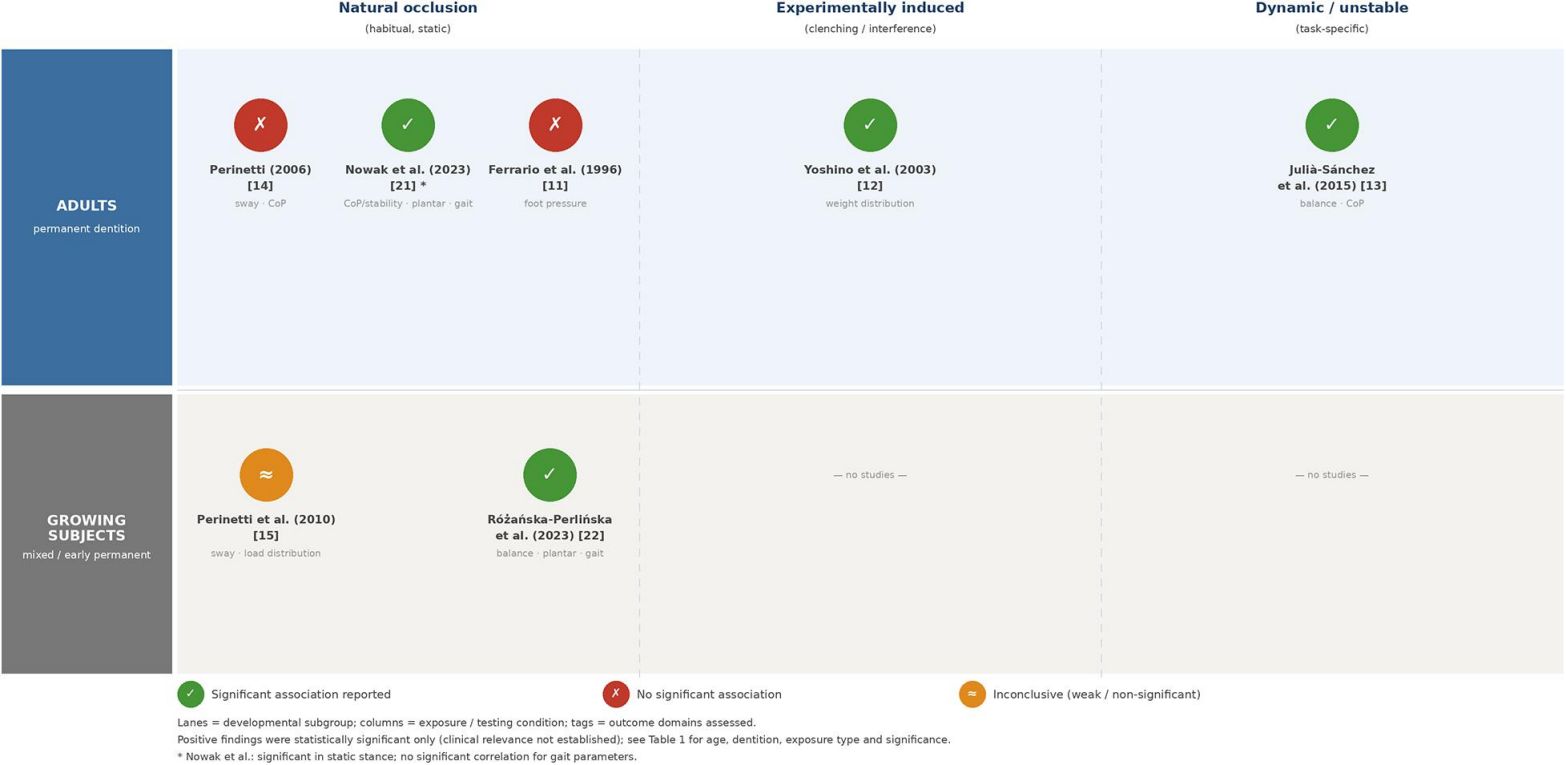
PRISMA 2020 flow diagram of the study selection process.

The electronic database search identified a total of 719 records. Specifically, 185 records were retrieved from PubMed/MEDLINE, 165 from Scopus, 158 from Embase, 112 from CINAHL, and 99 from Web of Science. In addition, two further records were identified through manual reference list screening and citation tracking. After removal of 291 duplicate records, 430 records were screened by title and abstract. Of these, 380 were excluded because they did not address the relationship between dental malocclusion or occlusal conditions and objective postural, balance, gait, or plantar pressure assessment, or did not meet the predefined inclusion criteria. Fifty full-text articles were assessed for eligibility, of which 43 were excluded. The main reasons for exclusion were absence of objective posturographic or plantar pressure assessment (*n* = 21), lack of analysis of malocclusion or occlusal condition as the primary exposure (*n* = 13), and inappropriate study design or population (*n* = 9). Finally, seven studies met all eligibility criteria and were included in the qualitative synthesis. The study selection process is summarised in the PRISMA flow diagram (Figure 1). A complete list of excluded studies together with the corresponding reasons for exclusion is provided in Supplementary Table S1.

### 3.2 Characteristics of included studies

The main characteristics of the seven included studies, together with the assessed postural and plantar outcomes, statistical approaches, and reported associations, are summarized in Table 1. All studies employed observational designs, primarily cross-sectional, and included human participants without neurological or musculoskeletal conditions that could interfere with postural assessment.

**Table 1:** Study characteristics, methodological features, postural and plantar outcomes, and reported associations with occlusal conditions of included studies.

Author (Year)	Country	Study design	Sample size (*n*)	Population/Age group	Dentition stage	Occlusal condition evaluated	Postural/plantar assessment	Postural condition	Postural condition assessed	Main postural/plantar variables	Statistical approach	Reported association (context-dependent)
Ferrario et al. (11)	Italy	Experimental (within-subject)	30	Adults	Permanent	Experimental occlusal interference	Force platform (center of foot pressure)	Static stance	Static	Center of foot pressure displacement	Within-subject comparison	Yes
Yoshino et al. (12)	Japan	Experimental (within-subject)	20	Adults	Permanent	Occlusal support loss during clenching	Force platform (weight distribution)	Static stance	Static	Weight distribution at the feet	Within-subject comparison	Yes
Perinetti (14)	Italy	Cross-sectional	55	Children / adolescents	Permanent	Natural dental occlusion	Stabilometric platform (CoP displacement, sway)	Static stance	Static	Postural sway, CoP displacement	Correlation analysis	No
Perinetti et al. (15)	Italy	Cross-sectional	281	Adults	Permanent	Sagittal and transverse malocclusion	Stabilometric platform (postural sway, load distribution)	Static stance	Static	Postural sway, weight distribution	Multiple regression	Inconclusive
Julià-Sánchez et al. (13)	Spain	Cross-sectional	30	Adults	Permanent	Dental occlusion under unstable conditions	Stabilometric platform (CoP, stability indices)	Dynamic/unstable platform	Dynamic (unstable platform)	Postural stability, CoP displacement	Between-condition comparison	Yes
Nowak et al. (21)	Poland	Cross-sectional	90	Adults	Mixed/early permanent	Occlusal asymmetry and malocclusion	Plantar pressure platform	Static stance	Static	Plantar pressure distribution, balance	Between-group comparison	Yes
Różańska-Perlińska et al. (22)	Poland	Cross-sectional	60	Adolescents (12–15 yrs)	Mixed/early permanent	Malocclusion (Angle classification)	Plantar pressure system + gait analysis	Static stance/gait	Static/gait	Static balance, plantar load symmetry; gait parameters	Between-group comparison	Yes

Adult participants were defined as individuals aged ≥18 years (predominantly 20–35 years) with established permanent dentition and no reported neurological or musculoskeletal conditions affecting postural control.

Growing subjects corresponded to participants aged 12–15 years, including mixed or early permanent dentition, as reported in the original studies

CoP, center of pressure. “Reported association (context-dependent)” refers to the presence of statistically significant relationships between occlusal conditions and postural or plantar outcomes under the specific assessment conditions defined in each study.

Sample sizes varied across studies. Occlusal conditions were defined using heterogeneous criteria, including sagittal discrepancies, transverse asymmetries, occlusal interferences, and experimentally induced occlusal alterations. These exposures comprised both naturally occurring malocclusion and transient experimental perturbations, which were considered separately in the narrative synthesis because they cannot be interpreted as equivalent clinical conditions. Despite this variability, all studies evaluated occlusal conditions as the primary exposure variable.

Postural assessment methods were objective in all cases and included stabilometric platforms and/or plantar pressure systems, enabling quantitative evaluation of postural control. Static posturography was used in all studies, while dynamic conditions or functional tasks were incorporated in a subset of investigations. Reported outcomes included center-of-pressure displacement, postural sway, weight-bearing asymmetry, and plantar pressure distribution (11–15, 21, 22).

### 3.3 Postural and plantar outcomes

**Figure 2:**
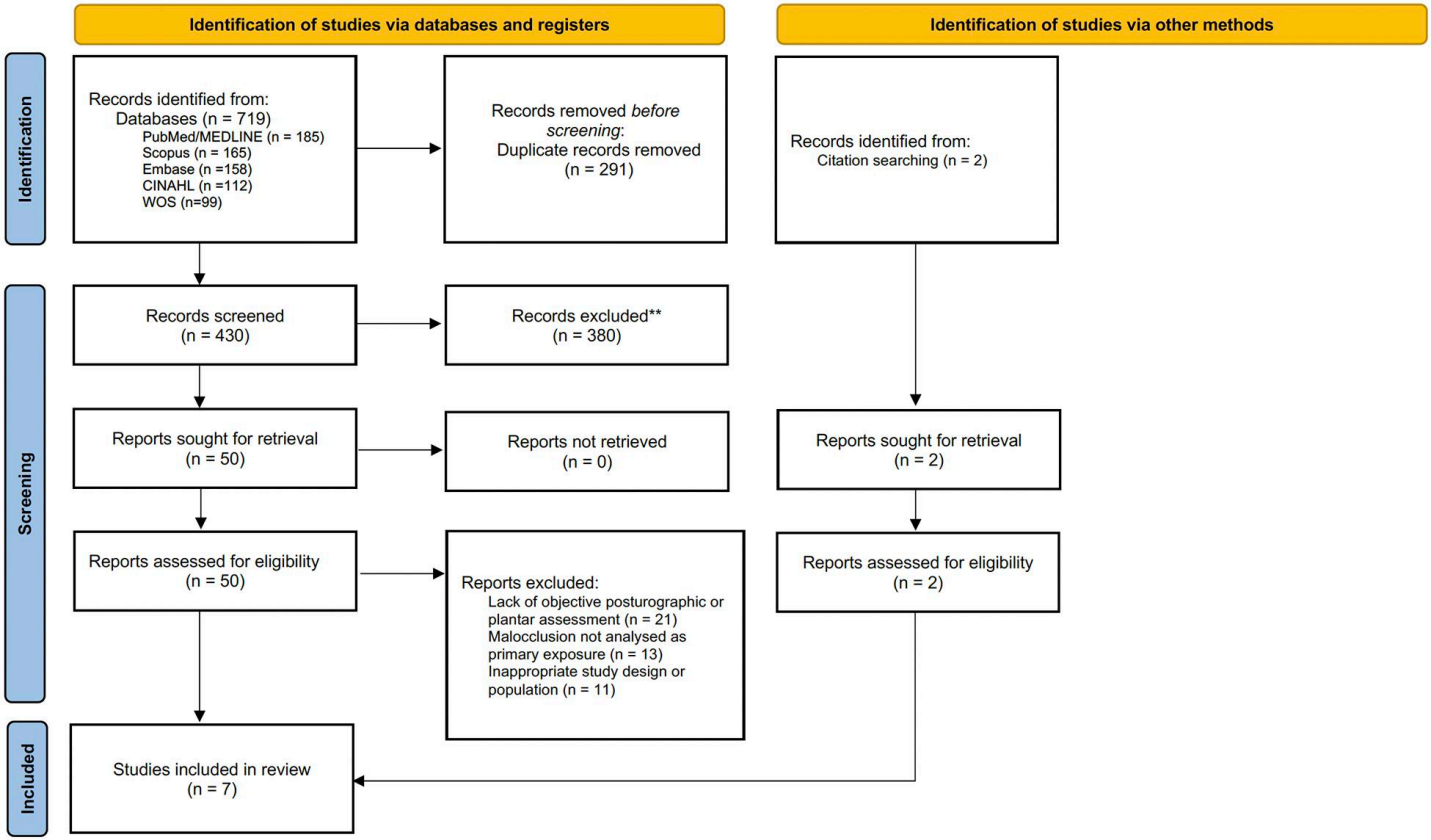
Overview of associations between occlusal conditions and postural outcomes stratified by developmental stage.

An overview of the postural and plantar domains assessed across the included studies, together with the variability in assessment protocols and reported associations, is summarized in Figure 2.

Given the physiological differences in postural control maturation, results were stratified by age group.

#### 3.3.1 Results in adult populations

Five studies evaluated adult subjects with fully established permanent dentition. Findings in this subgroup were inconsistent, with the majority of studies reporting no significant association between occlusal conditions and postural parameters under standard assessment conditions.

Specifically, the cross-sectional study by Perinetti (14), based on static stabilometric analysis, did not identify any correlation between occlusal characteristics and postural sway, whereas Nowak et al. (21) reported significantly higher static stabilographic parameters and a forward shift of the center of pressure in adults with malocclusion under natural conditions, with no significant correlation for gait parameters. Ferrario et al. (11), comparing adults with asymmetric (Angle Class II) malocclusion during natural standing, found no significant effect on center of foot pressure. The remaining studies reported changes only under specific conditions: Yoshino et al. (12) observed significant weight-distribution shifts only during active clenching, and Julià-Sánchez et al. (13) reported significant effects of occlusion on balance exclusively under dynamic or unstable conditions, with no differences in static assessments.

Overall, these findings indicate that, in adult populations, the association with natural occlusal conditions is inconsistent across studies, and that measurable postural responses emerge mainly under experimental or task-specific conditions. Findings obtained during clenching, artificially induced occlusal interferences, or unstable or task-specific testing should be distinguished from those obtained under routine, naturally occurring malocclusion, as the former reflect transient experimental perturbations rather than the habitual clinical condition.

#### 3.3.2 Results in growing subjects

Two studies evaluated growing subjects, defined as children and adolescents in the mixed or early permanent dentition with craniofacial growth and postural maturation not yet completed; the specific age range, dentition stage and developmental characteristics reported in each study are shown in Table 1. The two studies reported conflicting results. Perinetti et al. (15), in the largest sample (122 young subjects, 10.8–16.3 years) assessed by static posturography, found no meaningful correlation between malocclusion and postural sway, with only a few weakly significant associations. In contrast, Różańska-Perlińska et al. (22) reported associations between Angle classification and both static balance parameters and gait-related variables.

Given that the larger study was essentially null and the two studies disagree, the current evidence does not indicate a more consistent association in growing subjects, and any positive signal should be regarded as preliminary and hypothesis-generating rather than an established pediatric association.

### 3.4 Methodological quality and risk of bias assessment

The assessment of risk of bias is presented in Table 2.

**Table 2:** Methodological appraisal (JBI).

Author (Year)	Study design	JBI domain assessment	Participant selection & inclusion criteria	Measurement of exposure (malocclusion/occlusal condition)	Measurement of outcomes (postural/plantar assessment)	Identification & control of confounders	Appropriateness of statistical analysis	Overall risk of bias
Ferrario et al. (11)	Experimental observational	Moderate	Moderate	Moderate	Low	Moderate	Moderate	Moderate
Yoshino et al. (12)	Experimental observational	Moderate	Moderate	Moderate	Low	Moderate	Moderate	Moderate
Perinetti (14)	Cross-sectional	High	Low	Low	Low	Moderate	Low	Moderate
Perinetti et al. (15)	Cross-sectional	High	Low	Low	Low	Moderate	Low	Low–moderate
Julià-Sánchez et al. (13)	Cross-sectional	High	Low	Low	Low	Low	Low	Low
Nowak et al. (21)	Cross-sectional	High	Moderate	Low	Low	Moderate	Moderate	Moderate
Różańska-Perlińska et al. (22)	Cross-sectional	High	Moderate	Low	Low	Moderate	Moderate	Moderate

Risk of bias was assessed using the Joanna Briggs Institute (JBI) Critical Appraisal Checklist for Analytical Cross-Sectional Studies, applied according to the design of each included study. Domain-level judgements considered participant selection, measurement of exposure and outcomes, identification and control of confounding factors, and appropriateness of statistical analyses. Overall risk of bias reflects a qualitative synthesis across domains based on the reviewers' judgement and does not represent a standardized or quantitative JBI score.

Across studies, variability was observed in several domains. Concerns related to participant selection and inclusion criteria, as well as identification and control of confounding factors, were the most frequently identified limitations. In contrast, outcome measurement, based on objective posturographic or plantar pressure assessments, was generally considered robust across studies.

Overall, most studies presented moderate concerns in at least one domain, particularly related to confounding and selection bias.

### 3.5 Certainty of evidence

The overall certainty of the evidence, assessed using the GRADE approach as an overall body-of-evidence judgment, was judged to be low to very low across all evaluated outcomes (see Supplementary Table S2).

This rating reflects several recurring methodological constraints identified across the included studies. Most investigations were observational and showed limitations related to confounding and participant selection. In addition, findings were not consistent across studies, with variations observed according to population characteristics and assessment conditions. Differences in the definition of malocclusion, as well as in posturographic and plantar assessment protocols, further contributed to this variability.

Imprecision was also apparent, given the limited number of included studies and the variability in sample sizes.

Overall, these factors indicate a limited level of confidence in the available evidence across all evaluated outcomes.

## 4 Discussion

This systematic review evaluated the relationship between occlusal conditions and postural or plantar parameters using objective posturographic and plantar pressure methods. Overall, the available evidence does not demonstrate a consistent and reproducible association between occlusal conditions and global postural control across different populations and assessment protocols.

### 4.1 Interpretation of findings

The findings of this review indicate that the relationship between occlusal conditions and postural control is variable and context-dependent rather than uniform. In adult populations, static stabilometric findings were inconsistent: one study detected no association (14) while another reported significant static differences (21), and the remaining positive findings were concentrated under experimental or dynamic conditions (11–13).

In growing subjects, the two available studies disagreed: the larger study was essentially null (15), whereas the smaller one reported positive associations (22). Taken together, the evidence does not show a clear age- or maturation-related pattern. Although it has been hypothesized that occlusion–posture associations might be more evident during active growth, our findings do not support this, as the larger growing-subjects study was negative; the available evidence is too limited and heterogeneous to draw any developmental conclusion.

Importantly, these patterns should be interpreted in light of the methodological limitations identified across studies. Domains related to confounding and participant selection were frequently associated with moderate risk of bias, which may have influenced the observed variability in findings. Because most included studies relied on crude group comparisons or limited adjustment, residual confounding cannot be ruled out. Some statistically significant associations may therefore reflect differences in maturation, anthropometry, physical activity, foot posture, cervical posture or sensory status rather than an independent effect of occlusal conditions.

Furthermore, the certainty of the evidence was rated as low to very low according to the GRADE approach. This reflects the observational nature of the included studies, the inconsistency of findings, and the presence of imprecision due to limited sample sizes. As a result, the confidence in the observed associations remains limited.

Taken together, the available evidence suggests that postural responses to occlusal conditions are not consistently detectable under natural conditions and may depend on specific experimental or developmental contexts.

### 4.2 Comparison with previous literature

Previous systematic reviews have predominantly focused on cranio-cervical posture assessed through cephalometric or photographic methods, rather than on global postural control evaluated using objective posturographic or plantar pressure measurements. These studies have generally reported heterogeneous findings and limited clinical relevance, even when quantitative synthesis was attempted (17).

Similarly, broader narrative and interdisciplinary reviews have highlighted potential links between occlusion, posture, and the musculoskeletal system, but have included heterogeneous outcomes and study designs, limiting direct comparison with instrument-based postural assessments (18, 19). Additional studies evaluating specific occlusal interventions have reported favorable changes in postural stability and head posture. However, differences in study design, outcome measures, and assessment protocols limit direct comparison with the evidence synthesized in the present review. Taken together, these findings further suggest that any association between occlusal conditions and postural control is likely to be context-dependent and influenced by both methodological and population-related factors (16).

More recent evidence focusing on cranio-cervical relationships suggests that associations between malocclusion and head or cervical posture may be more consistently detected than those involving global postural control (23, 24). However, these findings are largely based on static and morphometric assessments and are subject to similar methodological limitations.

Overall, the progression of evidence suggests an increasingly refined understanding of the relationship between occlusion and posture. Earlier literature often proposed broad functional connections between the stomatognathic and postural systems, whereas subsequent studies highlighted substantial methodological heterogeneity and inconsistent findings. More recent investigations using objective posturographic and plantar pressure assessments have further clarified that any observed associations are likely to be context-dependent rather than universal, varying according to developmental stage, testing conditions, and the specific postural outcomes evaluated. This evolution in the evidence base supports a more cautious interpretation of the relationship between occlusal conditions and global postural control.

Within this context, the present review contributes by focusing exclusively on objective posturographic and plantar pressure outcomes. The findings indicate that associations observed at the cranio-cervical level do not necessarily translate into consistent alterations in global postural control, particularly in adult populations.

### 4.3 Clinical implications

From a clinical perspective, the available evidence does not support the use of occlusal characteristics as an isolated or reliable predictor of postural function. Although specific occlusal conditions or experimentally induced alterations may influence postural parameters under controlled conditions, these effects are not consistently observed under natural conditions.

Accordingly, assumptions regarding postural dysfunction based solely on occlusal findings are not justified. Clinical decision-making in orthodontics should therefore consider postural aspects within a broader, interdisciplinary framework and rely on objective assessment when clinically indicated. Specifically, current evidence does not support using malocclusion alone to diagnose postural dysfunction, nor providing orthodontic or occlusal treatment with the sole aim of improving posture, balance, gait, or plantar pressure.

### 4.4 Limitations and future directions

This review has several limitations. The number of included studies was limited, and their heterogeneity precluded quantitative synthesis. Variability in the definition of occlusal conditions, postural assessment protocols, and outcome measures reduced comparability across studies.

Furthermore, only studies published in English were included. Although this approach improved feasibility and consistency during study selection and data extraction, it may have introduced language and selection bias by excluding potentially relevant evidence published in other languages.

In addition, the predominance of observational designs and the moderate risk of bias in key domains limit the strength of causal inferences. The low to very low certainty of evidence further constrains the interpretability of the findings.

Several factors known to influence postural and plantar outcomes, including age, sex, body mass index, physical activity, foot posture, cervical posture, temporomandibular disorder symptoms, orthodontic history, visual and vestibular status, and other musculoskeletal conditions, were inconsistently reported and rarely adjusted for across the included studies. This limited control of confounding, also reflected in the risk-of-bias assessment, may inflate apparent associations and is a key reason for the low to very low certainty of the evidence.

Future research should prioritize standardized definitions of occlusal conditions, consistent posturographic protocols, and adequately powered study designs. Longitudinal and experimental studies are needed to clarify whether observed associations reflect causal relationships or context-dependent adaptations.

## 5 Conclusions

The available evidence suggests that the relationship between occlusal conditions and global postural control is inconsistent and highly dependent on developmental stage, experimental conditions, and assessment protocols when evaluated using objective posturographic and plantar pressure methods. Observed effects appear to depend mainly on specific experimental or testing conditions rather than on developmental stage. In growing subjects the two available studies were conflicting (one larger study null, one positive), so this evidence, with low to very low certainty, does not establish a pediatric association or support firm clinical inferences.

Given the low to very low certainty of the evidence and the methodological limitations identified across studies, current evidence does not support considering occlusal characteristics as an independent or clinically reliable determinant of postural function. Nor does it support diagnosing postural dysfunction from malocclusion alone or providing orthodontic or occlusal treatment solely to improve posture, balance, gait, or plantar pressure.

Future research should focus on standardized definitions of occlusal conditions, consistent posturographic protocols, and adequately powered study designs in order to clarify the clinical relevance of these interactions.

## Data Availability

The original contributions presented in the study are included in the article/Supplementary Material, further inquiries can be directed to the corresponding author/s.
