# Supplementary material for: Occlusal conditions, postural control and plantar parameters in adults and growing subjects: a systematic review of objective assessment methods
Source: Front Dent Med. 2026 Jul 9;7:1887418. doi: 10.3389/fdmed.2026.1887418 (PMC13391928; doi:10.3389/fdmed.2026.1887418)
Supplement: Supplementary file 1 [file Table1.docx]

**Supplementary Material 1**

GRADE assessment of certainty of evidence

| Outcome | Nº studies | Design | Risk of bias | Inconsistency | Indirectness | Imprecision | Explanation | Overall certainty |
| --- | --- | --- | --- | --- | --- | --- | --- | --- |
| Global postural control (static conditions) | 4 | Observational | Serious | Serious | Not serious | Serious | Conflicting results across studies; no consistent association in adult populations | Very low |
| Global postural control (dynamic conditions) | 2 | Experimental/observational | Serious | Moderate | Not serious | Serious | Positive findings mainly under experimental or unstable conditions | Low |
| Plantar pressure distribution | 2 | Observational | Serious | Serious | Not serious | Serious | Associations reported only in growing subjects; limited external validity | Very low |
