# Supplementary material for: Occlusal conditions, postural control and plantar parameters in adults and growing subjects: a systematic review of objective assessment methods
Source: Front Dent Med. 2026 Jul 9;7:1887418. doi: 10.3389/fdmed.2026.1887418 (PMC13391928; doi:10.3389/fdmed.2026.1887418)
Supplement: Supplementary file 2 [file Table2.docx]

**Supplementary Material 2**

Detailed search strategies for each database

The full electronic search strategies were developed to identify studies evaluating the association between dental malocclusion or occlusal conditions and objective measures of postural control or plantar function. The strategy combined controlled vocabulary (when available) and free-text terms related to both exposure and outcomes. Searches were adapted for each database according to its specific indexing system and syntax.

No restrictions on publication date were applied. Only studies published in English were considered eligible. The last search was conducted in October 2025.

**PubMed/MEDLINE**

(("Malocclusion"[Mesh] OR "Dental Occlusion"[Mesh] OR malocclusion OR "dental occlusion" OR occlusion OR "occlusal condition*" OR "occlusal interference*" OR "occlusal asymmetr*" OR "bite" OR "Angle classification")

AND

("Postural Balance"[Mesh] OR "Posture"[Mesh] OR posture OR "postural control" OR "postural stability" OR "balance" OR "body sway" OR "center of pressure" OR "centre of pressure" OR stabilometry OR posturography OR "plantar pressure" OR "foot pressure" OR gait))

**Scopus**

(TITLE-ABS-KEY(malocclusion OR "dental occlusion" OR occlusion OR "occlusal condition*" OR "occlusal interference*" OR "occlusal asymmetr*" OR bite OR "Angle classification")

AND

TITLE-ABS-KEY(posture OR "postural control" OR "postural stability" OR balance OR "body sway" OR "center of pressure" OR "centre of pressure" OR stabilometry OR posturography OR "plantar pressure" OR "foot pressure" OR gait))

**Web of Science**

TS=(malocclusion OR "dental occlusion" OR occlusion OR "occlusal condition*" OR "occlusal interference*" OR "occlusal asymmetr*" OR bite OR "Angle classification")

AND

TS=(posture OR "postural control" OR "postural stability" OR balance OR "body sway" OR "center of pressure" OR "centre of pressure" OR stabilometry OR posturography OR "plantar pressure" OR "foot pressure" OR gait)

**Embase**

('malocclusion'/exp OR 'dental occlusion'/exp OR malocclusion OR 'dental occlusion' OR occlusion OR 'occlusal condition*' OR 'occlusal interference*' OR 'occlusal asymmetr*' OR bite OR 'angle classification')

AND

('posture'/exp OR 'postural balance'/exp OR posture OR 'postural control' OR 'postural stability' OR balance OR 'body sway' OR 'center of pressure' OR 'centre of pressure' OR stabilometry OR posturography OR 'plantar pressure' OR 'foot pressure' OR gait)

**CINAHL**

((MH "Malocclusion" OR MH "Dental Occlusion" OR malocclusion OR "dental occlusion" OR occlusion OR "occlusal condition*" OR "occlusal interference*" OR "occlusal asymmetr*" OR bite OR "Angle classification")

AND

(MH "Posture" OR MH "Postural Balance" OR posture OR "postural control" OR "postural stability" OR balance OR "body sway" OR "center of pressure" OR "centre of pressure" OR stabilometry OR posturography OR "plantar pressure" OR "foot pressure" OR gait))

**Additional search methods**

- Reference lists of all included studies and relevant reviews were manually screened.
- Citation tracking was performed to identify additional eligible studies.
