# Supplementary material for: Occlusal conditions, postural control and plantar parameters in adults and growing subjects: a systematic review of objective assessment methods
Source: Front Dent Med. 2026 Jul 9;7:1887418. doi: 10.3389/fdmed.2026.1887418 (PMC13391928; doi:10.3389/fdmed.2026.1887418)
Supplement: Supplementary file 3 [file Table3.docx]

**Supplementary Table. Full-text articles assessed for eligibility and excluded (n = 43), classified by reason for exclusion.**

| **No.** | **Author (Year)** | **Title** | **Reason for Exclusion** |
| --- | --- | --- | --- |
| 1 | **Baldini et al. (2013)** | Evaluation of the correlation between dental occlusion and posture using a force platform | No objective posturographic or plantar pressure assessment |
| 2 | **Bracco et al. (2004)** | Effects of different jaw relations on postural stability in human subjects | No objective posturographic or plantar pressure assessment |
| 3 | **Cuccia & Caradonna (2009)** | The relationship between the stomatognathic system and body posture | No objective posturographic or plantar pressure assessment |
| 4 | **D'Attilio et al. (2004)** | The influence of an experimentally-induced malocclusion on vertebral alignment in rats | No objective posturographic or plantar pressure assessment |
| 5 | **De Wijer et al. (1996)** | Temporomandibular and cervical spine disorders: clinical signs and symptoms | No objective posturographic or plantar pressure assessment |
| 6 | **Dipalma et al. (2022)** | Dental occlusion and body posture: an overview | No objective posturographic or plantar pressure assessment |
| 7 | **Fink et al. (2002)** | The functional relationship between the craniomandibular system, cervical spine, and the sacroiliac joint | No objective posturographic or plantar pressure assessment |
| 8 | **Gangloff et al. (2000)** | Dental occlusion modifies gaze and posture stabilization in human subjects | No objective posturographic or plantar pressure assessment |
| 9 | **Gonzalez et al. (2008)** | Influence of temporomandibular joint dysfunction on muscle activity and posture | No objective posturographic or plantar pressure assessment |
| 10 | **Häggman-Henrikson et al. (2013)** | Head movements during chewing: relation to size and texture of bolus | No objective posturographic or plantar pressure assessment |
| 11 | **Huggare & Raustia (1992)** | Head posture and cervicovertebral and craniofacial morphology in patients with craniomandibular dysfunction | No objective posturographic or plantar pressure assessment |
| 12 | **Michelotti et al. (2011)** | The additional value of a home physical therapy regimen versus patient education only for the treatment of myofascial pain | No objective posturographic or plantar pressure assessment |
| 13 | **Monaco et al. (2011)** | Postural changes induced by unilateral chewing: a case-control study | No objective posturographic or plantar pressure assessment |
| 14 | **Munhoz et al. (2005)** | Influence of bilateral crossbite on the adaptation of the cervical spine | No objective posturographic or plantar pressure assessment |
| 15 | **Nicolakis et al. (2000)** | An investigation of the relationship between craniomandibular disorders and poor posture | No objective posturographic or plantar pressure assessment |
| 16 | **Olivo et al. (2006)** | Association between head and cervical posture and temporomandibular disorders | No objective posturographic or plantar pressure assessment |
| 17 | **Piancino et al. (2009)** | Chewing in unilateral crossbite patients before and after orthodontic treatment | No objective posturographic or plantar pressure assessment |
| 18 | **Sakaguchi et al. (2007)** | Examination of the relationship between mandibular position and body posture | No objective posturographic or plantar pressure assessment |
| 19 | **Tardieu et al. (2009)** | Dental occlusion and postural control in adults | No objective posturographic or plantar pressure assessment |
| 20 | **Tecco et al. (2010)** | Walking with a unilateral composite resin occlusal splint influences electromyographic activity of masticatory and leg muscles | No objective posturographic or plantar pressure assessment |
| 21 | **Valentino et al. (2002)** | Electromyographic activity in subjects with different vertical facial morphology | No objective posturographic or plantar pressure assessment |
| 22 | **Al-Ani et al. (2005)** | Stabilisation splint therapy for temporomandibular pain dysfunction syndrome | Malocclusion not the primary exposure variable |
| 23 | **Bonnier et al. (2002)** | Cervical posture and craniofacial morphology | Malocclusion not the primary exposure variable |
| 24 | **De Boever et al. (2000)** | Need for orthodontic treatment in patients with temporomandibular disorders | Malocclusion not the primary exposure variable |
| 25 | **Ferreiro Ranieri et al. (1996)** | Role of proprioception of the stomatognathic system in postural tone | Malocclusion not the primary exposure variable |
| 26 | **Gravante et al. (2003)** | Plantar pressure distribution in the normal human foot during standing | Malocclusion not the primary exposure variable |
| 27 | **La Touche et al. (2011)** | The influence of cranio-cervical posture on maximal mouth opening and pressure pain threshold | Malocclusion not the primary exposure variable |
| 28 | **Lippold et al. (2003)** | Relationship between thoracic, lordotic, and pelvic inclination and craniofacial morphology | Malocclusion not the primary exposure variable |
| 29 | **Manfredini et al. (2012)** | Dental occlusion, body posture and temporomandibular disorders: where we are now | Malocclusion not the primary exposure variable |
| 30 | **Moya et al. (1994)** | Relationship between dental occlusion and plantar arch | Malocclusion not the primary exposure variable |
| 31 | **Perillo et al. (2011)** | Orthodontics or orthopedics between the jaws and the spine: a retrospective study | Malocclusion not the primary exposure variable |
| 32 | **Perinetti et al. (2013)** | Postural stabilometric variations induced by dental occlusal stimulation: a systematic review | Malocclusion not the primary exposure variable |
| 33 | **Sforza et al. (2006)** | Postural adjustments in children with different angle classes | Malocclusion not the primary exposure variable |
| 34 | **Armijo-Olivo et al. (2010)** | Muscle activation of the temporomandibular and cervical region during EMG tasks | Inappropriate study design or population |
| 35 | **Bergamini et al. (2008)** | Dental occlusion and body posture: a surface EMG study | Inappropriate study design or population |
| 36 | **Biasotto-Gonzalez et al. (2008)** | Correlation between temporomandibular disorders, posture, and malocclusion | Inappropriate study design or population |
| 37 | **D'Amico (1958)** | The canine teeth: normal functional relation of the natural teeth of man | Inappropriate study design or population |
| 38 | **Gomes et al. (2010)** | Relationship between temporomandibular disorders and pressure pain threshold in patients with chronic pain | Inappropriate study design or population |
| 39 | **Iunes et al. (2009)** | Craniocervical posture analysis in patients with temporomandibular disorder | Inappropriate study design or population |
| 40 | **Milani et al. (2000)** | Relationship between dental occlusion and mandibular rest position | Inappropriate study design or population |
| 41 | **Passarelli et al. (2023)** | Systematic review on malocclusion and body posture | Inappropriate study design or population |
| 42 | **Silvestrini-Biavati et al. (2013)** | Clinical association between chewing side preference and lateral spinal curvature | Inappropriate study design or population |
| 43 | **Tartaglia et al. (2011)** | A review of research on facial growth and adult craniofacial structure | Inappropriate study design or population |
| 44 | **Venancio & Camparis (2002)** | How common is orofacial pain? A prevalence study | Inappropriate study design or population |

*Reasons for exclusion: (1) absence of objective posturographic or plantar pressure assessment (n = 21); (2) malocclusion not evaluated as the primary exposure variable (n = 12); (3) inappropriate study design or population, including secondary literature, case reports, conference abstracts, and animal or in vitro studies (n = 11).*
